# US Taxation of Gambling Winnings and Incentives to Bet

**DOI:** 10.1007/s10899-023-10189-z

**Published:** 2023-02-11

**Authors:** Karl Whelan

**Affiliations:** University College Dublin, Dublin, Ireland

**Keywords:** Sports betting, Gambling, Income tax

## Abstract

Sports betting is growing rapidly in the US after its legalization by the Supreme Court in 2018. This paper describes the treatment of gambling winnings and losses in the federal tax code and shows how the system may incentivize some gamblers to substantially increase the scale of their betting in order to have a chance to win. This incentive stems from the fact that gambling losses can only be deducted if taxpayers are filing for itemized deductions, meaning the scale of gambling losses has to be large enough to push a taxpayer’s eligible deductions over the standard deduction. This incentive to engage in large-scale betting applies mostly to lower and middle-income households.

## Introduction

It is well known that the tax treatment of investment gains and losses can have an important effect on risk-taking decisions. Classic contributions including Domar and Musgrave (1944), Feldstein (1969) and Poterba (2002). Research on gambling has also pointed to the role that taxation has played in determining the form and location of betting markets and in affecting the extent of losses experienced by different groups.^1^ This paper examines how the current US tax code affects the incentives for taking risk by betting on sports. This is an important topical issue because in May 2018, the United States Supreme Court declared the Professional and Amateur Sports Protection Act (PASPA) to be unconstitutional, thus opening the way for individual states to introduce legislation permitting sports betting.

**Fig. 1:**
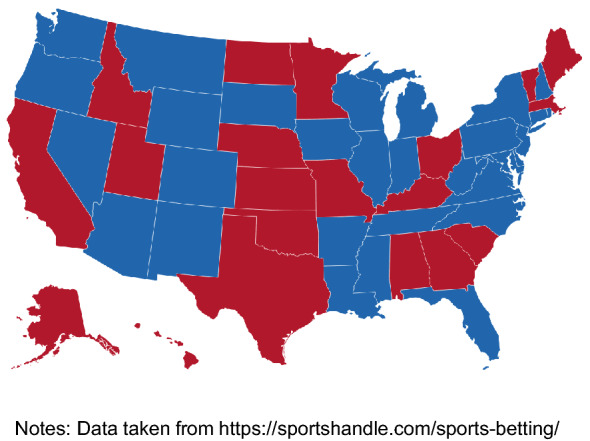
Legalized Sports Betting by States as of June 2022, Blue = Legalized, Red = Not Legalized. *Notes*: Data taken from https://sportshandle.com/sports-betting/

As of June 2022, thirty states had legalized sports betting in various forms and other states are likely to follow (see Fig. 1). Gambling in these markets is already large, with $136 billion placed in legal sports betting markets since the 2018 Supreme Court ruling but the market seems likely to grow substantially over the next few years.^2^ To give a potential benchmark, in the UK, which has a well-developed sports betting market, the total amounts of betting on sports per year is currently about £36 billion, equivalent to £530 per person.^3^ An equivalent amount of sports betting per person in the US would see annual totals of about $210 billion, equivalent to about 1% of current GDP or about four times the estimated size of the market in 2021.^4^

While many US states have welcomed the opportunity that legal sports betting gives them for earning additional revenues, the legalization of sports betting and its easy availability via cell phones is also likely to result in a significant increase in problem gambling. A 2021 review of the evidence by Public Health England estimated that one in two hundred of the British adult population had a problem with gambling and about one in twenty five were gambling at levels placing them “at risk”. Academic research such as Muggleton et al. (2021) has found that gambling raises the risk of financial distress and lowers health and well-being outcomes and there are active debates about how public policy can reduce gambling-related harm (King & Delfabbro, 2019).

Many of the issues with problem gambling in Europe stem from practices by bookmakers that encourage those who are losing to keep betting and discourage winners. Online gamblers are profiled and those who appear to be a good source of revenue are encouraged via free bets and special offers while those who tend to be winners find limits set on how much they can bet or else are banned altogether.^5^ The opportunity to capture a share of the lucrative emerging US market has attracted heavy involvement from European bookmakers, either via direct entry such as Paddy Power owner Flutter’s acquisition of FanDuel or via selling services to newly-licensed US bookmakers (known usually as sportsbooks) to allow them to copy the European business model.^6^

In light of the likely future social consequences of problem sports gambling in the US, this paper documents an unfortunate feature of the federal tax treatment of gambling that is likely to encourage some people to gamble large amounts of money relative to their incomes. Gambling winnings are taxed but gambling losses can only be deducted from income when the taxpayer is filing itemized tax deductions. For those who already have a large amount of itemized deductions, the taxation of net gambling winnings reduces their net gain but does not change how often they will come out ahead. For a standard bet in which a gambler bets $110 to potentially win $100, the required win rate to break even is 52.4% both when there is no tax and when a gambler is already claiming itemized deductions before gambling losses are factored in.

This means people who are reasonably well off—those with a large amount of state income taxes paid, or mortgage interest to deduct or charitable contributions to deduct—can approach their gambling in line with the advice on many websites and gambling guides that suggest you can make money if you can win more often than 52.4% of the time. However, most people that gamble in the US do not deduct their gambling losses. The federal tax system provides a large standard deduction—$12,950 for single people and $25,900 for married couples in 2022—so most people with gambling earnings are better off going for this option rather than itemizing. This makes it harder for them to win at gambling. For example, for the standard 110/100 bet and a marginal income tax of 22%, the required win rate to break even for those claiming the standard deduction is 58.5%.

This may seem like a relatively small increase in required winning percentage but this difference is crucial. It is hard enough for bettors to aim for winning 52.4% of the time—clearly, the average gambler wins 50/50 bets half the time—so a 58.5% winning rate against well-informed bookmakers is essentially impossible. However, depending on the level of potentially itemizable deductions that a person has, this breakeven win rate can be reduced a lot closer to the 52.4% rate provided the gambler chooses to “go big” with their gambling and itemizes their gross losses. We document the size of betting activity that would induce gamblers to believe they have a realistic chance of winning and it suggests an incentive has been created—largely for low and middle earners—to decide (like the Redditor quoted above) that their only chance of winning is to increase the scale of their betting.

The rest of the paper is organized as follows. Section 2 describes how federal income taxation of gambling works and how this interacted with the 2018 increase in the standard deduction. Section 3 illustrates how the various options open to taxpayers impact their chances of winning at sports betting. It describes how those who fall short of itemizing deductions without gambling losses may be incentivized to bet larger amounts. Section 4 concludes with some reflections on policy options.

## The US Federal Income Tax and Gambling Winnings

In addition to a 0.25% federal excise tax on all money wagered in gambling levied on gambling firms, money won via gambling is subject to federal personal income tax. For those who bet regularly, this federal income tax is hard to avoid. Sportsbooks are required by law to submit a Form 1099-MISC notifying the IRS of the total gross winnings of customers (the sum of all their winnings on those bets that won) once net winnings exceed $600 during a year.^7^ Gamblers can deduct their gross losses but only if they are itemizing deductions and these losses can only be used to offset gross winnings. You do not get a tax break for having net losses on gambling. Gambling winnings can also be subject to state-level taxes, with treatment varying across states.

In recent years, about 2 million federal income tax forms per year have declared gambling winnings. The total amount reported in 2019 was $35.8 billion. Most of these people did not itemize their losses because they were better off to claim standard deduction instead. The incentive to itemize fell in 2018 when the Tax Cuts and Jobs Act (TCJA) almost doubled the size of the standard deduction, from $6350 to $12,000 for single people and from $12,700 to $24,000 for married people. As shown in Table 1, the percentage of taxpayers claiming itemized deductions dropped from 30.6% in 2017 to 11.4% in 2018.^8^ Because many people with relatively small amounts of potentially itemizable deductions switched to claiming the standard deduction, the average size of itemized deductions increased for most income groupings, see Table 2. The exception to this pattern was very high income groups, whose deductions fell because TJCA introduced caps on the amount of state taxes and mortgage interest that could be deducted.

**Table 1:** Gambling winnings and itemized deductions

Year	Fraction of returns with itemized deductions (%)	Fraction of returns with gambling winnings (%)	Average of gambling winnings
2013	30.1	1.3	$15,629
2014	29.6	1.3	$15,763
2015	29.6	1.3	$17,044
2016	30.0	1.3	$15,987
2017	30.6	1.4	$15,558
2018	11.4	1.3	$16,512
2019	11.0	1.2	$18,359

Data from various editions of the IRS Statistics of Income Complete Report

**Table 2:** Changes in itemized deduction patterns from 2017 to 2018

	2017		2018	
Adjusted gross income	Percent of returns with itemized deductions (%)	Average itemized deduction	Percent of returns with itemized deductions (%)	Average itemized deduction
Under $20,000	5	$15,648	1	$21,963
$20,000 to $30,000	11	$16,596	3	$23,518
$30,000 to $40,000	16	$16,786	4	$23,091
$40,000 to $50,000	25	$16,737	7	$23,533
$50,000 to $75,000	37	$18,489	11	$23,301
$75,000 to $100,000	53	$21,139	17	$25,824
$100,000 to $200,000	76	$26,322	27	$30,564
$200,000 to $500,000	93	$44,137	48	$39,100
$500,000 or more	93	$211,562	70	$147,118
All	31	$29,926	11	$37,010

Data from IRS (2017, 2018)

Those with gambling earnings are more likely to itemize than others but, among this group, the percentage itemizing deductions also fell from 53% in 2017 to 27% in 2018. See Table 3. There are a number of reasons why those with gambling incomes are more likely to itemize deductions. First, as illustrated in Tables 4 and 5, the fraction of tax forms reporting gambling earnings rises with income and higher-income taxpayers are more likely to itemize deductions. Second, the ability to deduct gambling losses may make it more profitable for some taxpayers to itemize.

**Table 3:** Itemization of gambling winnings and losses

Year	Fraction of returns with gambling earnings that itemize deductions (%)	Average of gambling winnings when itemizing deductions	Average of gambling losses itemized
2013	54.1	$24,544	$20,927
2014	52.8	$25,361	$21,542
2015	52.7	$27,970	$23,923
2016	53.4	$25,204	$22,296
2017	52.9	$25,401	$23,072
2018	26.6	$46,669	$41,159
2019	28.4	$49,007	$44,442

Data from various editions of the IRS Statistics of Income Complete Report

**Table 4:** Gambling earnings and losses for 2017

Adjusted gross income	Fraction of returns with gambling earnings (%)	Average gambling earnings reported	Fraction of returns with gambling earnings itemizing deductions (%)	Average gambling winnings with itemized deductions	Average gambling losses deducted when deductions are itemized
Under $20,000	0.7	$4312	13	$6949	$6105
$20,000 to $30,000	0.9	$4705	26	$7635	$6209
$30,000 to $40,000	1.0	$4,387	33	$6866	$5565
$40,000 to $50,000	1.4	$6586	39	$9847	$9160
$50,000 to $75,000	1.6	$6199	51	$8950	$7617
$75,000 to $100,000	2.1	$7192	60	$8618	$7821
$100,000 to $200,000	2.4	$11,565	81	$12,973	$11,691
$200,000 to $500,000	2.5	$32,615	96	$33,814	$27,998
$500,000 or more	2.7	$349,333	95	$354,239	$253,873
All	1.4	$15,558	53	$25,401	$20,193

Data from IRS (2017)

**Table 5:** Gambling earnings and losses for 2018

Adjusted gross income	Fraction of returns with gambling earnings (%)	Average gambling earnings reported	Fraction of returns with gambling earnings itemizing deductions (%)	Average gambling winnings with itemized deductions	Average gambling losses deducted when deductions are itemized
Under $20,000	0.7	$3846	5	$5345	$4767
$20,000 to $30,000	1.0	$4711	9	$13,038	$12,978
$30,000 to $40,000	1.0	$4908	11	$16,108	$15,970
$40,000 to $50,000	1.0	$4933	20	$10,124	$8918
$50,000 to $75,000	1.5	$6103	24	$10,527	$10,304
$75,000 to $100,000	2.0	$8291	29	$17,944	$14,849
$100,000 to $200,000	2.2	$11,675	37	$23,429	$21,433
$200,000 to $500,000	2.6	$36,513	66	$48,991	$43,582
$500,000 or more	2.7	$137,034	83	$369,727	$270,290
All	1.3	$16,512	27	$46,669	$37,951

Data from IRS (2018)

Comparing Table 2 with Tables 4 and 5, we can see that for each level of income, the fraction of those with gambling winnings that itemize deductions is higher than for other taxpayers. This is particularly true of middle-income people. For example, of those with adjusted gross income of between $50,000 and $75,000, 29% of taxpayers reporting gambling earnings itemized deductions in 2018, compared with 11% among all taxpayers in this income bracket. Comparing Tables 4 and 5, we can see the fraction of gamblers itemizing deductions fell in 2018 after the increase in the standard deduction but the overall pattern of itemization relative to the rest of the public remained about the same.

**Fig. 2:**
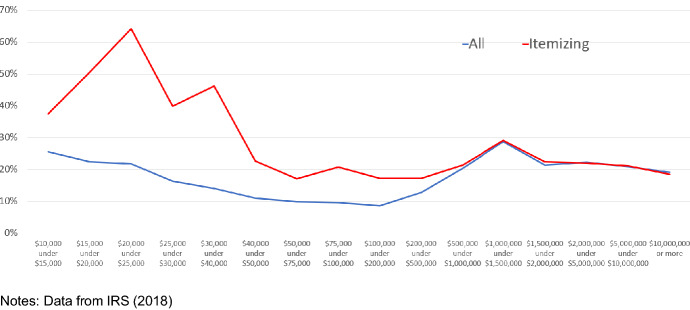
Average gambling earnings as a percent of adjusted gross income for all returns with gambling earnings and for those with gambling earnings and itemized deductions

A few other aspects of Tables 4 and 5 are worth noting. While most people don’t gamble, the average amounts risked by those who do are large. The average amount of annual gambling winnings reported was $16,512 in 2018, while the amount of winnings reported by those who itemized deductions jumped from about $25,000 in 2017 to about $47,000 in 2018 because many with smaller amounts of gambling losses decided to switch from itemizing to taking the standard deduction. These large average amounts are not simply due to rich high rollers who can afford to gamble lots of money. Tables 4 and 5 show high average levels of gambling earnings relative to income for each category. Figure 2 charts the ratio of average gambling winnings to average adjusted gross income for the most detailed income categories reported by the IRS. Average gambling earnings do tend to rise with income but, for all income ranges, average reported gambling winnings were around 10% or more of average adjusted gross income. For those itemizing, this ratio is even higher. In particular, for those with incomes under $40,000, the ratio of average gambling earnings to average adjusted gross income is over 40% for gamblers who itemize deductions.

These figures are significant because they show that, even prior to the widespread legalization of sports betting and the huge amount of advertising that has accompanied it, some gamblers were willing to put a large amount of their income at stake and to use itemized deductions as part of their betting strategy. Since it is unlikely these gamblers did better on average than the 4.5% loss that a typical bettor will incur on toss-up bets with 110/100 odds, the total amount of bets placed is likely to be twice as high as the reported earnings. This suggests many gamblers who itemized deduction typically placed bets worth over 40% of their adjusted gross income and often much more.

Finally, it is worth clarifying that the 20% average gap between reported winnings and reported losses shown in Tables 4 and 5 does not mean that gamblers with itemized deductions made net profits from their gambling. For each individual tax form, the maximum amount of losses that can be deducted is the amount of gross winnings. So, by definition, the sum of reported losses cannot exceed the sum of reported winnings. In fact, a simulation can show that the observed 20% difference between reported winnings and reported losses is consistent with a large number of betters each making 10 toss-up bets per year with 110/100 odds. These hypothetical gamblers would lose 4.5% on their bets on average before tax but their tax forms would show average reported winnings 20% above their average reported losses.

## Federal Income Taxes and Win Rates for Betting

Here we derive formulas for the net payoff from betting once taxation is taken into account. We then provide some numerical examples of how these calculations work in practice.

### After-Tax Net Payoffs and Breakeven Rates

First consider the case without taxation. A gambler wins a fraction *p* of their bets. When they do so, they receive a payout of *O* for a $1 bet, meaning the profit on a $1 bet is $$O - 1$$. Averaging across a large amount of bets, the net expected amount of winnings per $1 bet will be

1 $$\begin{aligned} p \left( O - 1 \right) - \left( 1 - p \right) = p O - 1 \end{aligned}$$

The break-even win rate condition for making positive expected profits is

2 $$\begin{aligned} p \ge \frac{1}{O} \end{aligned}$$

Now consider a marginal income tax at rate $\tau$ on gambling winnings.^9^ We will consider three cases. First, a gambler that will claim itemized deductions on their tax form, independent of their gambling losses, because they have a total *I* of eligible non-gambling-related deductions and *I* is greater than the standard deduction of *D*. This person will use their gambling losses to offset their gambling winnings. If they win more than they lose, then they pay tax on their net winnings. If they lose more than they win, they can offset the losses against the wins, so their after-tax net loss is the same as their pre-tax net loss. Their net after-tax return on gambling an amount *B* plus their tax relief from deductions is

3 $$\begin{aligned} \Omega _1 = \left\{ \begin{array}{llll} \left( 1- \tau \right) \left( p O - 1 \right) B + \tau I &{} \text {if} &{} p O \ge 1 \\ \left( p O - 1 \right) B + \tau I &{} \text {if} &{} p O < 1 \\ \end{array}\right. \end{aligned}$$

To give an example, if someone bets $1000 and wins $550 and loses $450, they will retain $100$$\left( 1-\tau \right) $$ after tax. If they win $400 and lose $600, they will not be charged taxes and their net losses will be the same as their gross loss of $200. In this case, taxes introduce an asymmetry in payoffs and mean smaller take-home winnings for the gambler but the break-even win rate of $$p = \frac{1}{O}$$ is the same as if there were no taxes.

Second, consider a gambler that chooses to use the standard deduction of *D* when filing their federal taxes. They do this because the sum of their gambling losses and other potential itemizable deductions, *I* are less than *D*. In other words

4 $$\begin{aligned} \left( 1 - p \right) B + I \le D \end{aligned}$$

The combined level of expected after-tax gambling winnings and tax relief from the standard deduction for this bettor will be

5 $$\begin{aligned} \Omega _2 = B \left( p \left( 1 - \tau \right) \left( O - 1 \right) - \left( 1 - p \right) \right) + \tau D \end{aligned}$$

The break-even probability condition for making positive expected profits from gambling after taxes are accounted for is now

6 $$\begin{aligned} p \ge \frac{1}{\tau + \left( 1 - \tau \right) O} \end{aligned}$$

Third, consider the case where a gambler’s potential itemized deductions are not enough on their own to make it worthwhile filing with itemized deductions but whose gross gambling losses are large enough to make itemizing worthwhile. In this case

7 $$\begin{aligned}&I < D \end{aligned}$$

8 $$\begin{aligned}&\left( 1 - p \right) B + I \ge D \end{aligned}$$

If pre-tax net expected winnings are positive ($p O > 1$) so all losses can be deducted, then the combined level of expected after-tax gambling winnings and tax relief will be

9 $$\begin{aligned} \Omega _3 = \Omega _2 + \tau \left( \left( 1 - p \right) B + I - D \right) \end{aligned}$$

If pre-tax net winnings are negative, then their deduction is limited to the their gross winnings so

10 $$\begin{aligned} \Omega _3 = \Omega _2 + \tau \left( p B + I - D \right) \end{aligned}$$

Simplifying the algebra, the combined level of after-tax gambling winnings and tax relief becomes

11 $$\begin{aligned} \Omega _3 = \left\{ \begin{array} {lll} \left( 1 - \tau \right) \left( p O - 1 \right) B + \tau I &{} \text {if} &{} p O \ge 1 \\ \left( p O - 1 \right) B + \tau I &{} \text {if} &{} p O < 1\\ \end{array}\right. \end{aligned}$$

This individual will break even on the combined return from betting activities and the additional tax relief relative to taking the standard deduction as long as

12 $$\begin{aligned} p \ge \frac{1}{O} + \frac{\tau }{1-\tau } \frac{D - I }{ B O} \end{aligned}$$

This breakeven probability lies in between the level with no taxes (Eq. 2) and the level with taxes and the standard deduction (Eq. 6). The larger the volume of betting *B* and the closer the person is to already having itemized deductions equal to the standard deduction (the smaller is $D-I$), the closer the break-even win probability gets to the tax-free equivalent rate.

The evidence suggests that the first of the three cases here, where $I > D$, only applies to about 10% of tax forms and these people will tend to have high incomes. We know that most gamblers do not itemize, so most of them will be consistent with the second case, where $I < D$ and $\left( 1 - p \right) B + I \le D$. However, the scale of betting activity is something that gamblers get to decide themselves. It may be that some people will conclude they are better off to bet large amounts to improve their breakeven rate.

### Numerical Examples

In providing specific examples, we will focus on the typical bet made by an American sports gambler in which $110 is placed to earn $100 in profit (equivalent to $$O = 1.91$$). We could also consider other longer or short-odds bets but the logic would not change.

The 110/100 bet usually involves a spread where the team perceived as weaker has been given some additional scores to make the adjusted game a toss up with equal chances of either sides of the bet winning. The gap between the $110 placed and the potential $100 in winnings represents the bookmaker’s margin (also known variously as the hold, the vigorish or “the juice”.) One way to think of this margin is to imagine the bookmaker taking in $220 in bets on both teams in a game and paying out $210 to the winner while keeping the other $10. This represents a 4.5% gross profit rate for the bookmaker before they cover taxes and costs. Perhaps more usefully for us, 4.5% is the average pre-tax loss that will be incurred by a gambler that wins 50% of these bets, half the time winning $100 and the other half losing $110.

Without income tax, the value of $O = 1.91$ implies a break-even win percentage of $p = 0.524$. This win rate of 52.4% is well known to sports bettors and there are plenty of guides to betting that emphasize the need to win this much to overcome the bookmaker’s margin.^10^ But once income tax is considered, the breakeven win percentage rises. For the 22% tax rate paid by typical middle-income households, the breakeven win percentage rises to 58.5% if gambling losses are not itemized. For the highest-income taxpayers, it is 63.6%. See Table 6. These high breakeven win rates seem likely to discourage many potential gamblers from taking up sports betting.

Still, some gamblers could respond to this increased breakeven win percentage by shrugging it off, figuring their sporting acumen will be sufficient to eke out the extra few points of winning percentage need to offset the impact of taxes. They would be wrong. There is an enormous literature on the efficiency of sports betting odds, and while there is some evidence of mis-pricing of longshot bets in a way that generates greater losses for those who take those bets, overall the evidence suggests the probabilities implied by bookmakers odds closely track with actual win rates. So the average gambler wins toss-up 110/100 bets half the time. Getting win percentages higher than this is extremely difficult.

Ed Miller and Matthew Davidow’s (2019) fascinating book, *The Logic of Sports Betting*, describes various strategies that can be used to win at sports betting—most notably focusing on “exotic” bets and obscure markets where prices are not updated as often—but makes clear that implementing such strategies are very time-consuming and that win rates of 53% to 54% are as high as even the best-informed strategy can achieve. These win rates are below most of the realistic breakeven rates just calculated. The most famous gambler in the world, Tony Bloom, runs the secretive firm StarLizard which takes money from millionaires to bet on sports based on complex statistical algorithms. Reports suggest that StarLizard look to make average profit margins of 1–3% on their bets.^11^ Starlizard bets in Asian markets which do not deduct tax and have low bookmaker’s margins, so it seems likely that this firm’s win rate could be as low as 52%. We can be sure that at most a tiny fraction of the new era of American sports bettors could match these win rates over an extended period.

Accepting that gamblers will generally do no better on average than win half their bets, Table 6 illustrates how income tax can hugely increase the average net loss from gambling when there are no itemized deductions. With a win rate of fity percent, even those paying the lowest federal income tax rate of 10% will see their expected loss rate increase to 9% while those on the top marginal tax rate would on average lose 21% on their bets.

**Table 6:** Breakeven win rates for taxpayers not itemizing deductions (2019 tax rates and percentages of taxpayers)

Marginal tax rate	Adjusted gross income		Percentage of taxpayers (%)	Breakeven win percentage (%)	Loss rate with 50% win percentage (%)
	Single	Married			
Zero	Zero	Zero	21.7	52.4	4.5
0.1	$0 to $9950	$0 to $19,900	16.3	55.0	9.1
0.12	$9951 to $40,525	$19,901 to $81,050	34.4	55.6	10.0
0.22	$40,526 to $86,375	$81,052 to $172,750	19.3	58.5	14.5
0.24	$86,376 to $164,926	$172,751 to $329,850	6.0	59.1	15.5
0.32	$164,926 to $209,425	$329,851 to $418,850	0.8	61.8	19.1
0.35	$209,426 to $523,600	$418,851 to $628,300	0.9	62.9	20.5
0.37	$523,600 or more	$628,300 or more	0.6	63.6	21.4

**Fig. 3:**
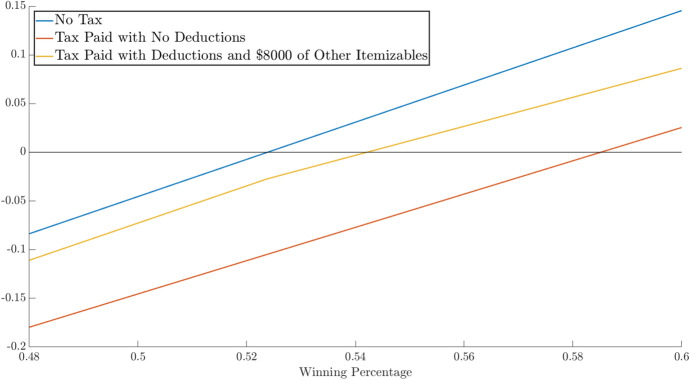
Net return by win percentage on a $1 bet without tax, with tax and standardized deduction and with tax and itemized deductions and $40,000 in bets placed ($$\tau = 0.22$$)

What about those who have non-gambling potentially itemized deductions below the standard deduction ($$I < D$$) but who could itemize if their gross gambling losses are large enough? Figure 3 illustrates the net return on a $1 bet as a function of win rates for three cases: No income tax, income tax and standardized deductions and income tax and itemized deductions with $40,000 in bets placed and $8000 in non-gambling itemizable deductions. We are assuming the gambler is single, so their standard deduction equals its 2022 rate of $12,950 and their marginal tax rate is assumed to be 22%. Relative to taking the standard deduction, the outcome per dollar risked is better for the gambler that placed $40,000 in bets and they break even with a 54% win rate.

**Fig. 4:**
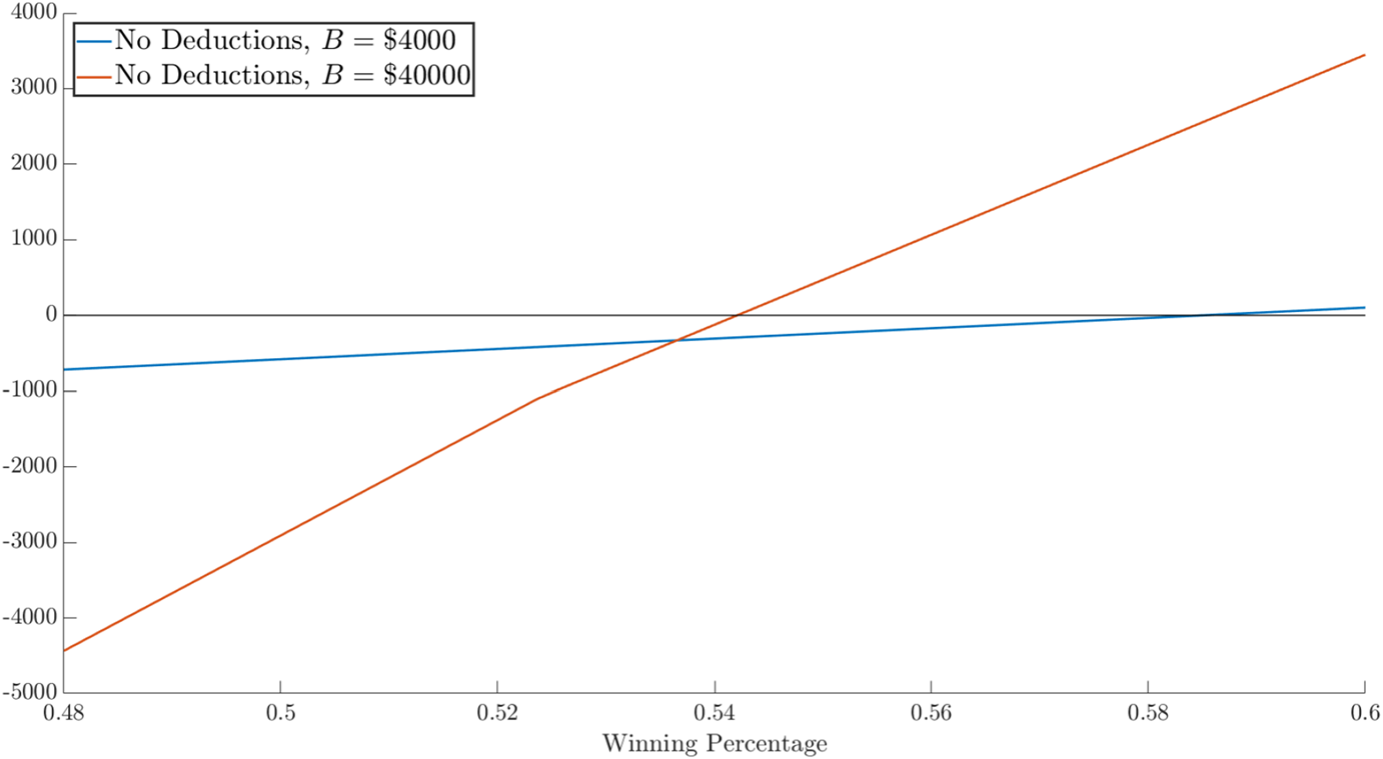
Net profit by win percentage for $4000 bet with no itemization and $40,000 bet with itemization and $$I = \$8000$$ ($$\tau = 0.22$$)

Figure 4 also illustrates why gamblers may think they are better off “going big.” It compares the net after-tax profits from betting $4000 per year and claiming the standard deduction with the outcome from betting $40,000 with $8000 in non-gambling itemizable deductions. At win rates of 54% and above, the gambler is better off with the big betting strategy. The flip side of the big betting strategy is that at more realistic win rates, the gambler is likely to lose about $3000 per year.

**Fig. 5:**
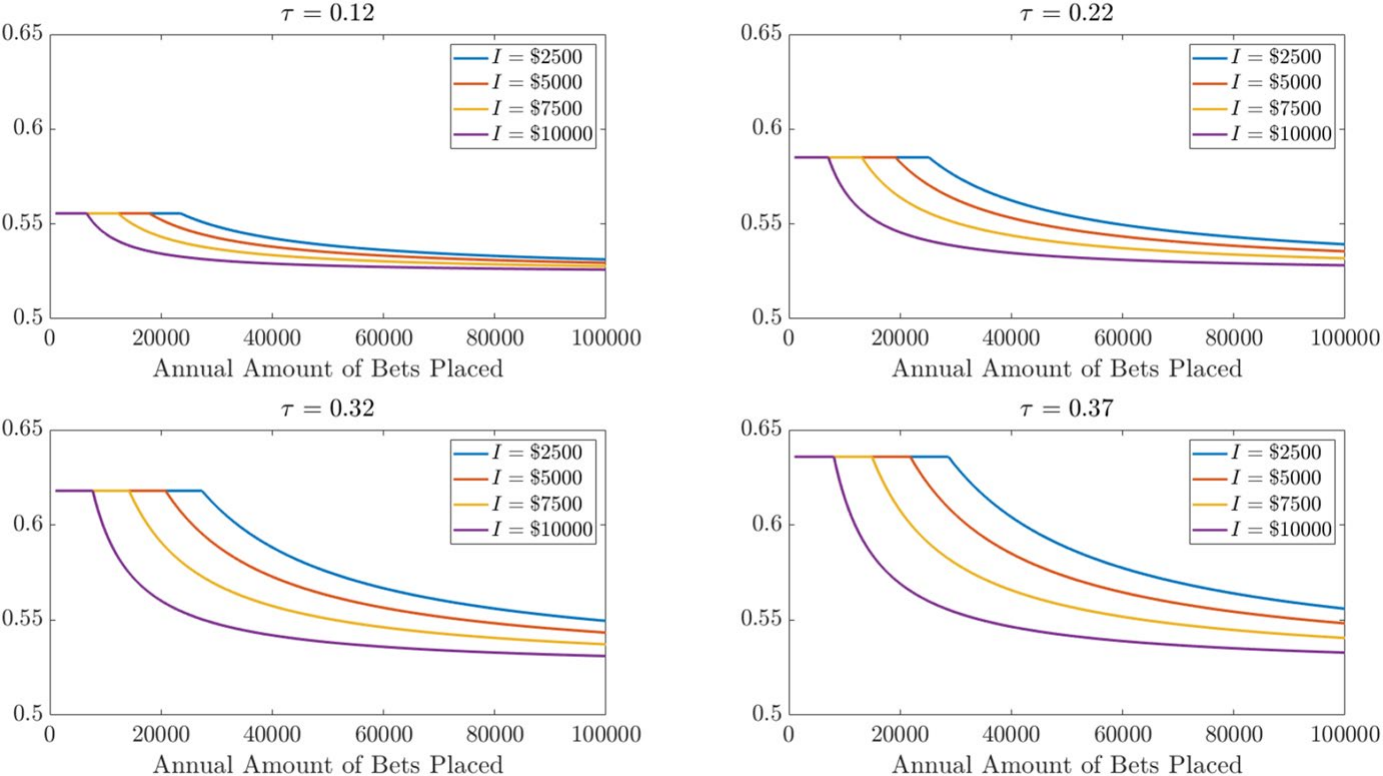
Breakeven win probabilities for total amounts bet and non-betting potential itemized deductions

Figure 5 shows how breakeven win rates depend on the marginal tax rate and on the extent of non-gambling-related items that can be itemized. Breakeven rates increase with marginal tax rates but, for each tax rate, the more other itemizable items there are, the smaller the amount of betting required to start reducing breakeven rates below those when claiming the standard deduction. Once the size of betting reaches the point where itemizing is better than the standard deduction, the breakeven win rates tend to fall quite quickly but, in general, the scale of betting required to get breakeven win rates to “realistic” (in the minds of some gamblers) levels of 53–54% is very large, particularly for those without many other itemizable deductions.

**Fig. 6:**
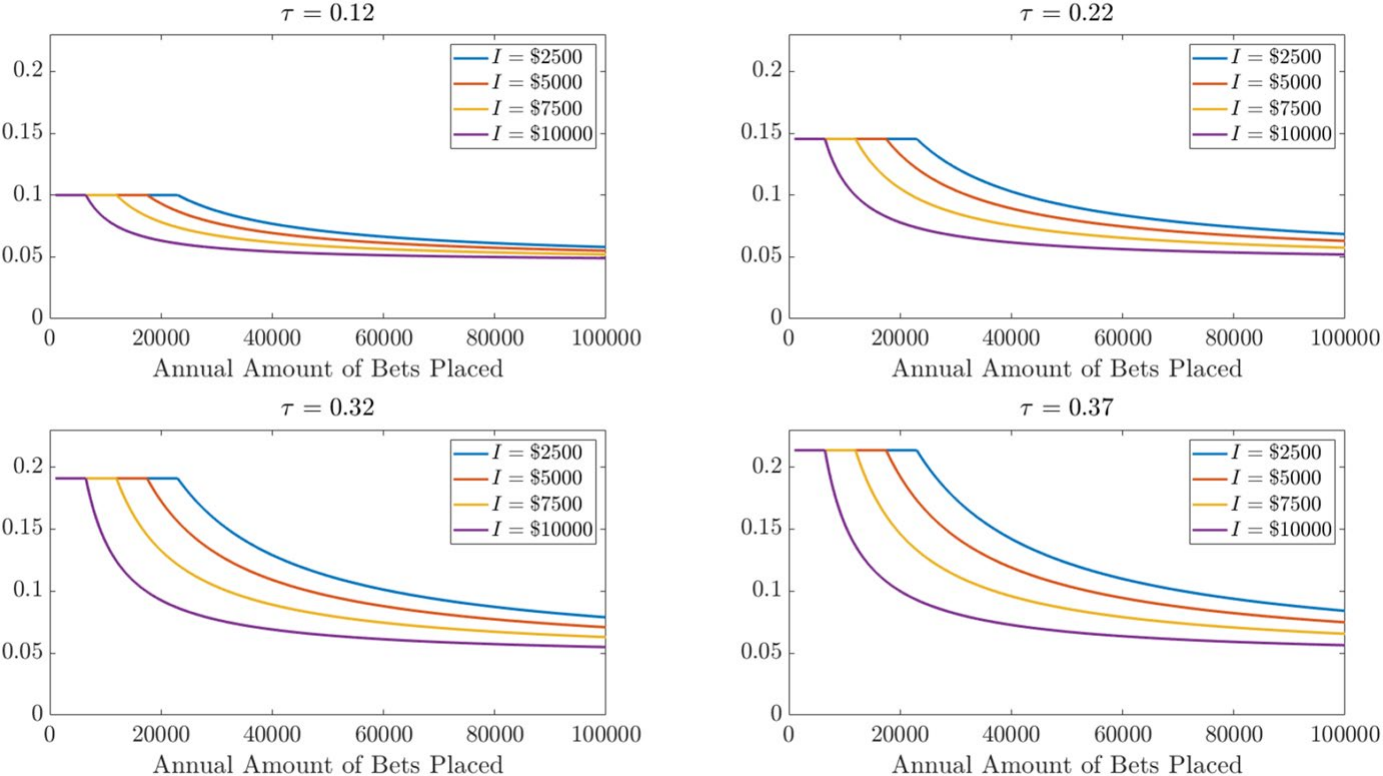
Expected loss rates for total amounts bet and non-betting potential itemized deductions when gambler wins 50% of bets

**Fig. 7:**
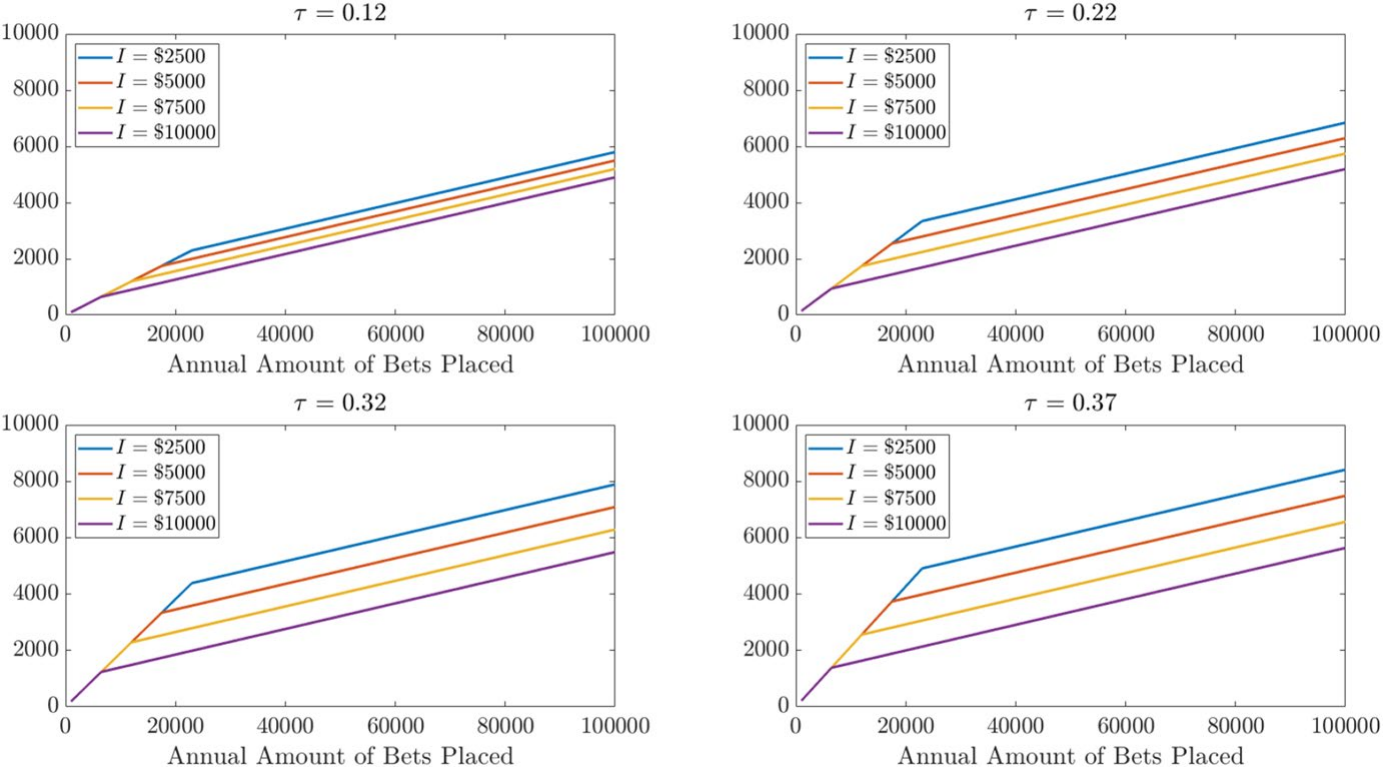
Expected loss amounts for total amounts bet and non-betting potential itemized deductions when gambler wins 50% of bets

Figure 6 illustrates the expected loss per $1 of betting associated with these different sets of parameters when the win rate is 50%. Loss rates fall rapidly once the scale of betting allows the gambler to itemize. But losses are still losses, and asymptoting towards losing 4.5% still generates big losses when a lot of money is risked. Figure 7 shows the total dollar amount of losses generated in each of these scenarios.

These calculations suggest the main source of concern in relation to the incentive to gamble large amounts is likely to be with low to middle earners, particularly the large numbers of people who switched from itemizing in 2017 to not itemizing in 2018. These individuals pay low marginal tax rates, making “reasonable” win percentages seem more within reach and, if they already have some non-gambling items to itemize, they may consider gambling large amounts to increase their chances of breaking even.

## Discussion

Given the growing evidence base showing that online sport betting can lead to many negative social consequences, what are going to be the likely consequences of the treatment of winnings and losses in the new sports betting industry in the US Federal Income tax code?

Over the next few years, one issue that will arise is that some people will continue to place bets without understanding the consequences for their tax liabilities. The leading sportsbooks in the US do provide information on the tax treatment of gambling winnings but it is not prominently displayed and discussions in online forums about sports betting suggest there is a widespread lack of understanding of the issue. This is exacerbated by the fact that while the requirements for sportsbooks to file forms notifying the IRS of winnings are relatively stringent, the requirements for the sportsbooks to withhold taxes are far less so. The widely-reported problems in the UK involving people who have gambled away large amounts of money could end up being even more severe in the US after gamblers are presented with big tax bills even though they have already lost plenty of money. The problems related to these information deficits could be improved by state government legislation to require sportsbooks to be clear about the tax consequences of betting and by changing IRS guidelines to require more withholding of tax on winnings.

Over the longer-run, there are different arguments about the impact of the taxation of gambling winnings and losses on the future scale of the US sports betting industry and on the extent of problem gambling. On the one hand, federal income taxation of winnings will make average net losses on sports betting much higher than in countries where gambling winnings are untaxed such as the UK. This tax is more visible to betters than taxes applied to sports betting in other countries, which tend to tax turnover or profits of bookmakers, and the incidence falls fully on the gamblers rather than being split between the gamblers and the bookmakers.

It may be that once most American sports bettors understand that the tax treatment of gambling winnings makes it almost impossible to come out ahead, they will be sufficiently discouraged that the emerging US sports betting market ends up being much smaller on a per capita basis than the UK market, thus limiting the extent of problem gambling. The evidence from the UK presented by Paton et al. (2004) suggests that sports betting volumes are highly sensitive to tax changes. As such, the US approach could be considered a Pigouvian tax that discourages something that is socially unproductive.

An alternative argument is that legalized sports betting will still attract many people who think (most likely incorrectly) they can come out ahead despite the tax treatment of winnings. In this case, while taxation of winnings may reduce the numbers that bet on sports, the scale of financial problems for those who do bet regularly could be worse than in the UK, potentially creating a greater issue with problem gambling. Certainly, the companies that currently dominate the US sportsbook market believe the industry is going to grow rapidly. This can be seen in the current scale of sportsbook advertising, which is now hugely prominent in US televised sports. Forbes have reported that total advertising spending by US sportsbooks was likely around $1.8 billion in 2022 and is projected to grow to $2.9 billion by 2024.^12^ To give a sense of why the sportsbooks consider this advertising to be worthwhile, DraftKings reported in 2020 that they spent an average of $371 per customer acquired in advertising costs and other costs such as free bets and they expected the average lifetime value of each customer to be $2500.^13^ This suggests the industry does not view federal taxation of gambling winnings as something that will substantially restrict its growth. The US will likely provide some interesting “natural experiments” on the impact of taxation on sports betting in the coming years because different states are going to take different approaches to applying state-level income taxes to betting winnings and to taxing the profits of sportsbooks.

Beyond the question of the extent to which gambling winnings should be taxed, there is surely a strong argument against the federal tax code encouraging people to think their best chance of winning at sports betting is to risk large amounts of money, particularly when these perverse incentives are going to apply most to low and middle-income people. In this respect, there are two options. The first is to treat only net gambling winnings as taxable income. This removes any incentive to bet larger amounts so as to avail of itemized deductions of losses. However, it could still be argued that this approach encourages higher betting amounts: Losses can only be used to offset winning, so once a gambler has won money, the tax deductibility of losses reduces net after-tax losses. For example, someone who has winnings of $100 and pays tax at a marginal tax rate of 24% will view the potential loss on another $100 bet as being $76 rather than $100.

The other option is to remove gambling losses as a deduction altogether. This may be the best option in terms of restricting the growth of problem betting in the US but it is likely to be politically difficult. The state-by-state process of obtaining licenses has involved the new US sportsbook businesses engaging in intense political lobbying, including making political contributions and providing hospitality to law-makers.^14^ This will likely be matched in future years by extensive lobbying at federal level, particularly if legislation aimed at reducing sports betting were to be proposed.

## Conclusions

While opportunities to gamble legally in the United States have been relatively restricted until recent years, we have documented that those who did gamble often placed large amounts of money at risk. The legalization of sports betting means that problem gambling is likely to become a more serious issue in the US over the next few years. Evidence from elsewhere corroborates this concern. The structure of the emerging US sports betting industry looks a lot like what has been in place in the UK in recent years. Indeed, some of the bookmakers that have a large share of the UK market have either entered the US sportsbook market directly or are providing services to newly-licensed US sportsbooks to operate models similar to the typical UK approach. The UK government has conducted a major review of the sports gambling industry and has concluded that it is doing a lot of harm. A bill setting out policy actions stemming from the review is due to be published soon.

In light of the evidence from the UK, the US should carefully consider its approach to taxation of gambling winnings. In this paper we have documented that the current system provides an incentive for some bettors, in particular those on low to middle incomes, to scale up the size of their betting activity to improve their chances of breaking even. It is recommended that the federal tax code be amended to remove this perverse incentive.

## Data Availability

All data used in the paper are publicly available from US Internal Revenue Service publications. I can supply the data on request.

## Footnotes

1 Roukka and Salonen (2020) describe the regressive impact of taxation of gambling in Finland.

2 Data from https://www.legalsportsreport.com/sports-betting/revenue/.

3 Sports betting revenue figures taken from https://www.gamblingcommission.gov.uk/statistics-and-research/publication/industry-statistics-november-2021.

4 The American Gaming Association estimates total sports betting placed in 2021 at $57 billion.

5 See Davies (2022a, b).

6 Hill (2019) is an informative and entertaining account of how the arrival of European bookmakers was changing the face of sports betting only one year after the repeal of PASPA.

7 Based on discussions in online forums, there seems to be a lot of confusion among gamblers about the tax treatment of their winnings and sportsbooks don’t seem to be keen to point out the tax implications. But if you dig deeply enough into the fine print, you will find admissions that they are reporting your winnings to the IRS. See this for example. https://support.fanduel.com/s/article/Taxes-Frequently-Asked-Questions.

8 All data reported here have been taken from various editions of the IRS’s *Statistics of Income* reports.

9 We will not consider the case here that taxable winnings could be large enough to cause someone’s marginal tax rate band to increase. In practice, this isn’t likely to be a common problem.

10 See Culver (2018).

11 See this story by Business Insider https://www.thejournal.ie/tony-bloom-starlizard-2597458-Feb2016/.

12 https://www.forbes.com/sites/bradadgate/2022/09/15/sports-betting-is-revving-up-ad-spending-for-fourth-quarter/.

13 https://frontofficesports.com/newsletter/sports-betting-primer/.

14 See the November 2022, New York Times story “Cigars, Booze, Money: How a Lobbying Blitz Made Sports Betting Ubiquitous”. Available at https://www.nytimes.com/2022/11/20/business/sports-betting-lobbying-kansas.html.
